# Bedside assessment of left atrial pressure in critical care: a multifaceted gem

**DOI:** 10.1186/s13054-022-04115-9

**Published:** 2022-08-13

**Authors:** Emma Maria Bowcock, Anthony Mclean

**Affiliations:** Intensive Care Unit, Derby Street, Nepean, Penrith, Sydney, 2747 Australia

**Keywords:** Left atrial pressure, Left atrial physiology, Left ventricular end-diastolic pressure, Transpulmonary circuit, Left atrial strain, Right ventricular–pulmonary circuit, Cardiac phenotypes

## Abstract

Evaluating left atrial pressure (LAP) solely from the left ventricular preload perspective is a restrained approach. Accurate assessment of LAP is particularly relevant when pulmonary congestion and/or right heart dysfunction are present since it is the pressure most closely related to pulmonary venous pressure and thus pulmonary haemodynamic load. Amalgamation of LAP measurement into assessment of the ‘transpulmonary circuit’ may have a particular role in differentiating cardiac failure phenotypes in critical care. Most of the literature in this area involves cardiology patients, and gaps of knowledge in application to the bedside of the critically ill patient remain significant. Explored in this review is an overview of left atrial physiology, invasive and non-invasive methods of LAP measurement and their potential clinical application.

**Graphical abstract:**

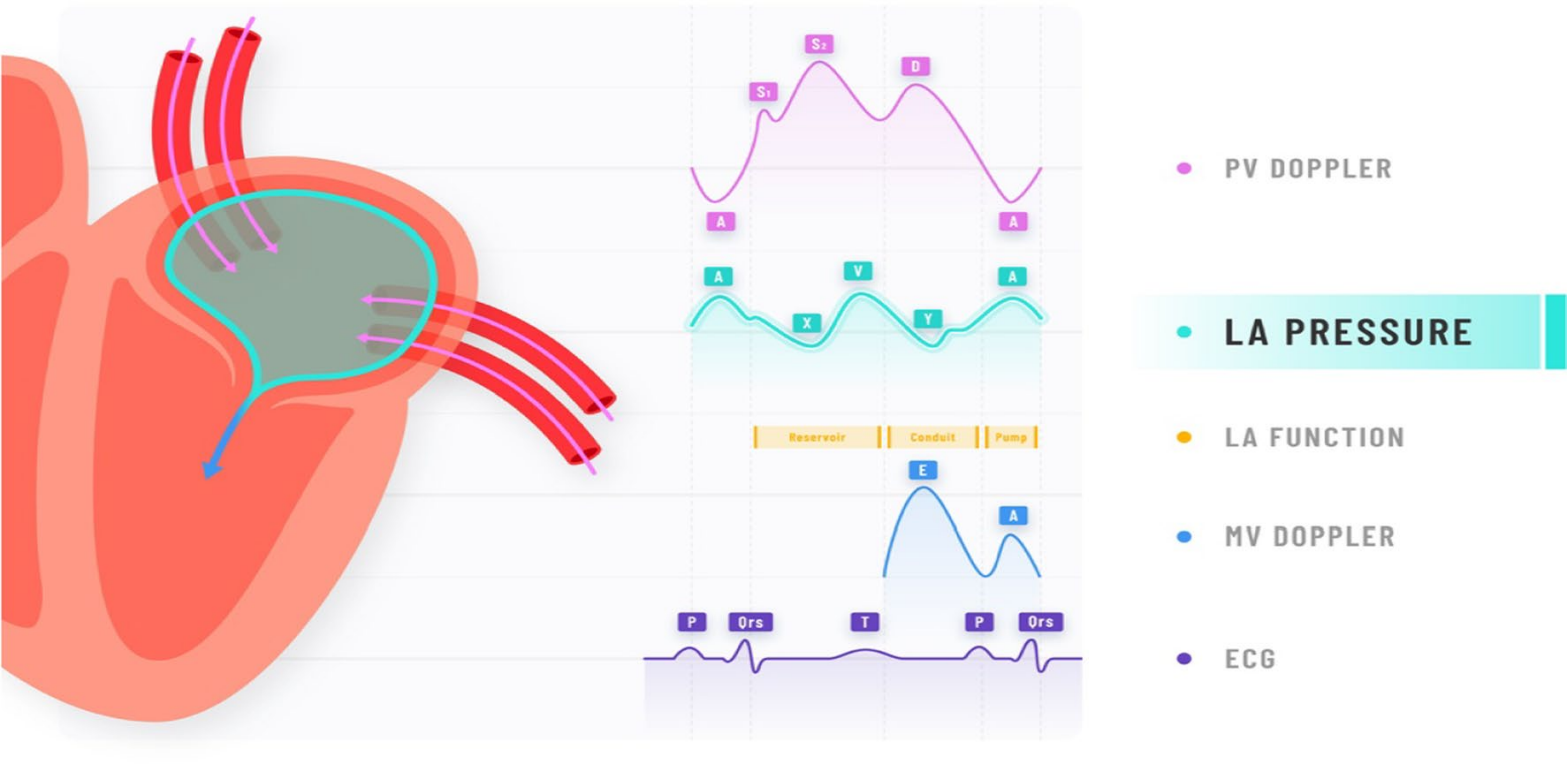

## Background

A clinician’s interest in the left atrial pressure (LAP) usually pivots around its preload contribution to cardiac output. However, the left atrium is a key component of the ‘transpulmonary circuit’ with upstream and downstream functions as reservoir, conduit and pump [1]. Increases in LAP have important consequences for gas exchange, pulmonary haemodynamic load and right ventricular performance [2]. Raised LAP may be due to pre-existing left ventricular systolic and/or diastolic dysfunction, mitral and/or aortic valve pathology; however, acute increases in LAP can be seen in critical illnesses such as sepsis, myocardial ischemia, stress-induced cardiomyopathies and volume overload states [3–5]. Accurate manipulation of cardiopulmonary performance using the limited tools available demands a more in-depth understanding of LA physiology and pressure measurement.

### Left atrial physiology

**Fig. 1:**
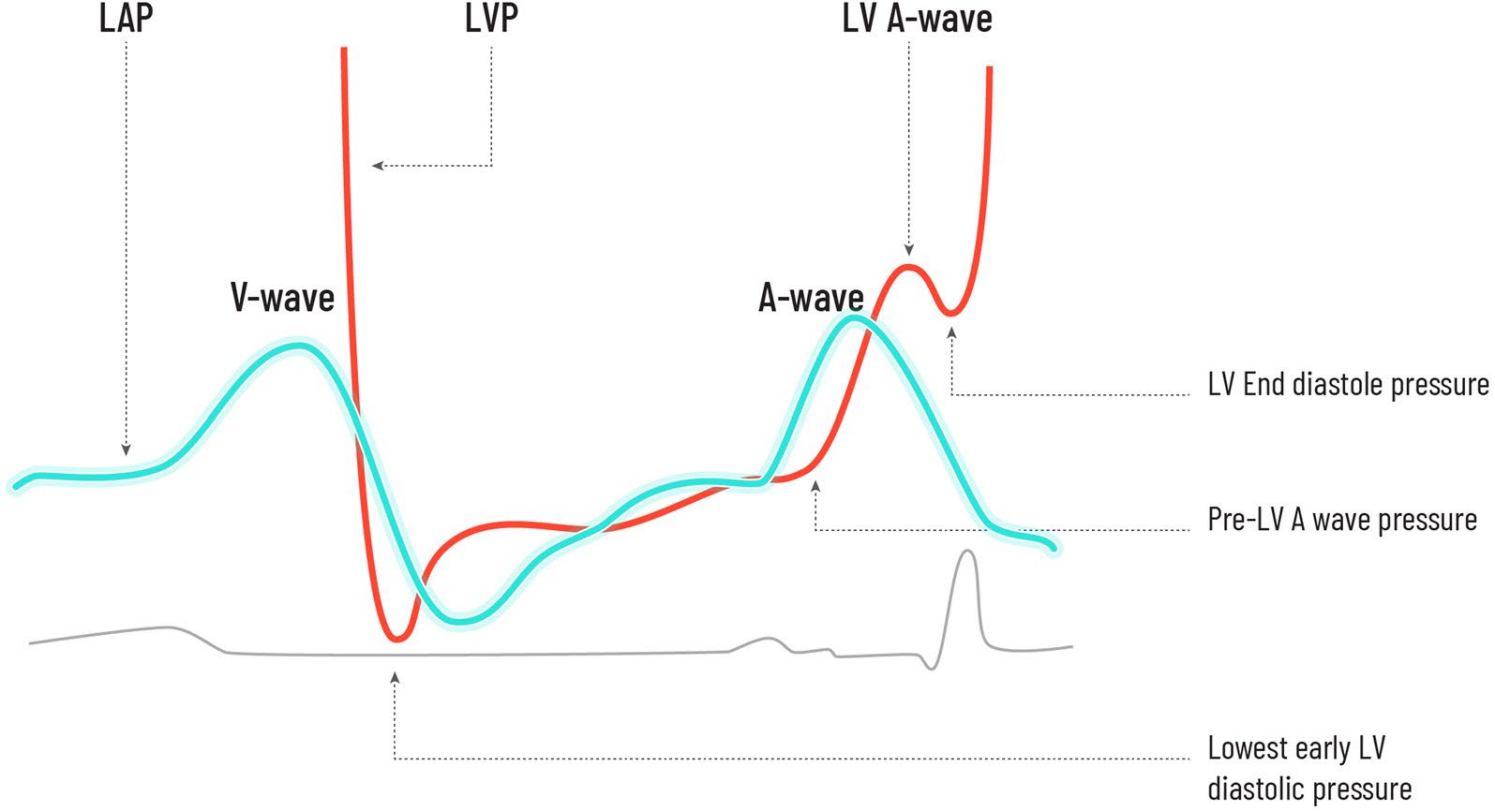
Relationship between the left atrial and left ventricular pressures

Although the classical anatomy is that of four pulmonary veins, two superior and two inferior, draining separately into the left atrium (LA), this is only the case in 70% of individuals [6]. Around 12–25% of the population have either the two right, or the two left pulmonary veins entering through a single ostia [6]. Flow from the pulmonary veins into the left atrium is pulsatile, and the classical pressure wave form exhibits a V wave and an A wave. The V waves are passive atrial filling waves and occur during ventricular systole. The other peak, the A wave, is the left atrial pressure wave that follows active atrial contraction [7, 8]. The relationship between the left atrial pressures and left ventricular pressures is illustrated in Fig. 1.

**Fig. 2:**
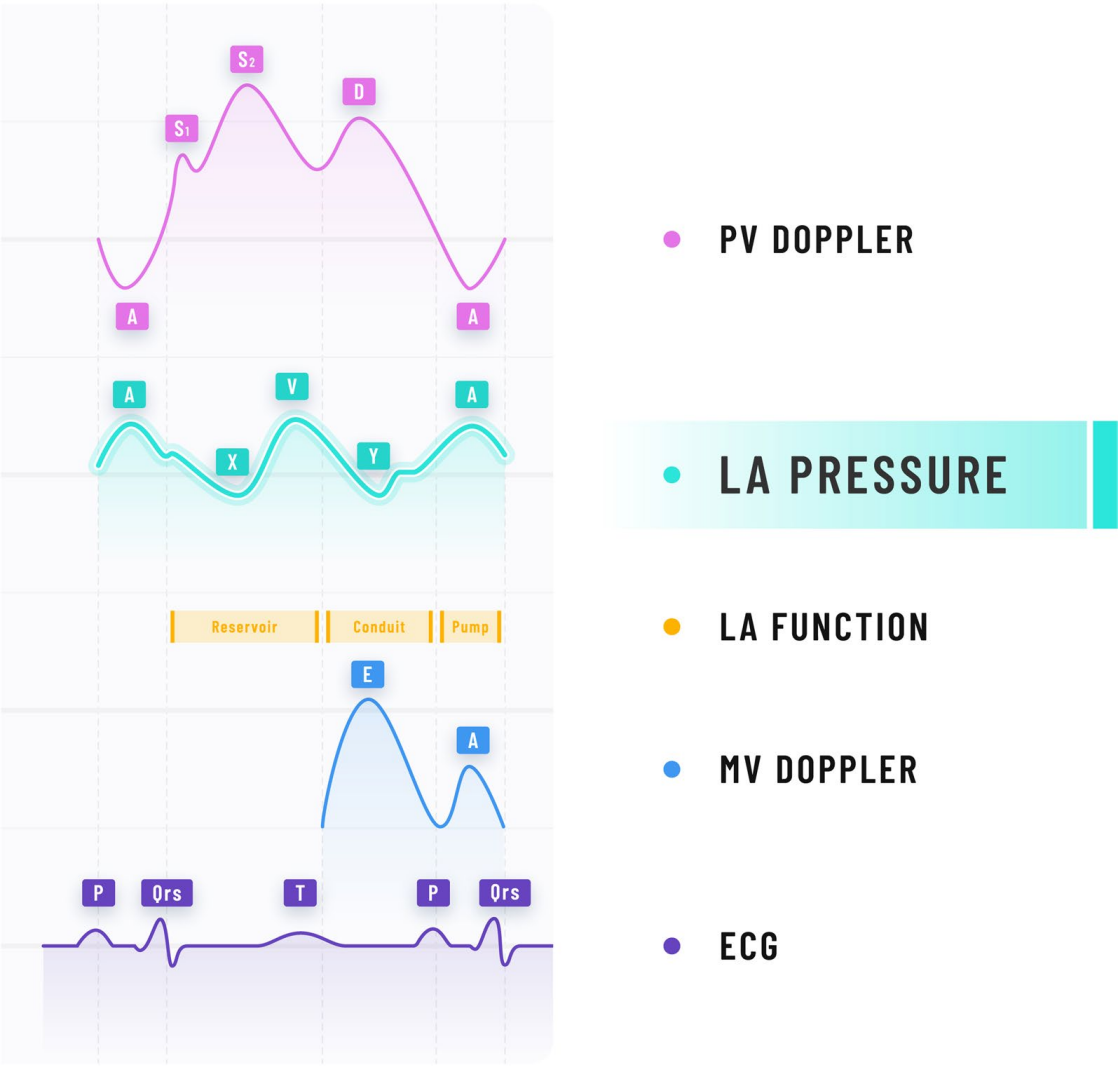
Relationship between pulmonary vein (PV) pressure, LAP and mitral inflow Doppler waves throughout the cardiac cycle. PV Doppler D wave mirrors the mitral E wave and occurs at the time of the Y descent. PV A wave is concomitant to the mitral Doppler A wave and to left atrial contraction. The corresponding reservoir, conduit and pump functions of the left atrium are shown. *MV* mitral valve

Blood flow from the pulmonary vein into the LA depends upon the pressure gradient, which varies throughout the cardiac cycle, i.e. the normal blood flow is both phasic and bidirectional [7]. Doppler analysis reveals four distinct waves of flow [8]. See Fig. 2. Two antegrade waves occur during the LA reservoir phase in early and mid-systole (S_1_ and S_2_, respectively), corresponding to the X descent post-A pressure wave. The V pressure wave caused by ventricular contraction reduces antegrade flow but following this during the Y descent comes the third antegrade flow during diastole, giving the pulmonary vein D wave, whose amplitude and shape mirror that of the mitral Doppler E wave. Near the end of diastole, atrial contraction occurs, resulting in a significant pressure difference between the LA and pulmonary vein creating a retrograde A wave into the pulmonary vein. This pulmonary vein Doppler A wave is related in time to the transmitral Doppler A wave and the LA pressure A wave [7, 8].

#### What are we measuring and why?

As demonstrated in Fig. 1, there is variation throughout the cardiac cycle and the pressure at a specific time point has consequences for both incoming flow from the PV (downstream) into the LA and ongoing flow from the LA into the left ventricle (LV). It is quite difficult to express LV filling pressure (LVFP) as a single value on the LV and LA pressure tracing because the pressures fluctuate and LV filling is a complex process.

Mean LAP and LVEDP are not telling us the same thing yet are often used interchangeably. The LVEDP provides information about the LV operating compliance and is the closest estimate of LV preload as a surrogate for LVEDV. Patients with similar LVEDP can have markedly different LAP, which is determined by the operating compliance of the LA [9]. This concept is perhaps most relevant to critical care as changes to compliance can occur with fluid challenges and mechanical ventilation for example. The *mean* LAP integrates the atrial pressure tracing throughout systole and diastole providing a measure of the hemodynamic load determined by the LA operating compliance (and indirectly left ventricular operating compliance through atrioventricular coupling). It is the *mean* LAP that is reflected back to the pulmonary venous circulation impacting right ventricular performance [9, 10].

**Fig. 3:**
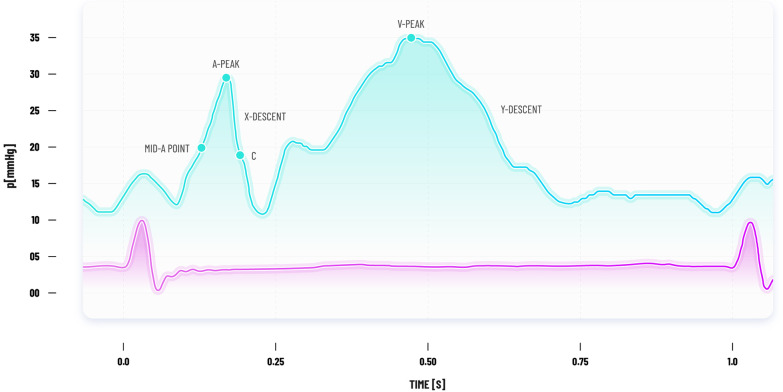
PAOP trace showing the ‘mid A point’ and large ‘V’ wave (patients with mitral regurgitation or reduced LA compliance). An integrated digitised mean over the entire cardiac cycle would include the ‘V’ wave and give a higher PAOP value than a PAOP measurement taken at the ‘mid A point’.* PAOP* pulmonary artery occlusion pressure

The ‘mid A wave pressure’ (mean value of the A‐wave amplitude) is recommended in consensus statements to estimate *end-diastolic* LAP that correlates most closely with LVEDP [11], whereas the *mean* LAP is obtained by temporal integration of the instantaneous PAOP over the entire cardiac cycle (Fig. 3). *Mean* LAP and *end-diastolic* LAP can differ significantly in the presence of large ‘V’ waves that occur in severe mitral regurgitation and with reduced LA compliance [12] (Fig. 3). Some suggest that the *mean* LAP as opposed to the *end-diastolic* LAP makes more sense when wanting to differentiate pre- from post-capillary pulmonary hypertension (PH) [9, 10]. Certainly, in the critically ill patient with hypoxic respiratory failure and RV dysfunction the more crucial question must be what the cumulative haemodynamic load on the pulmonary vascular system is. The answer to this lies with measurement of the *mean* LAP.

#### LAP and ‘RV–pulmonary circuit’ dysfunction

**Fig. 4:**
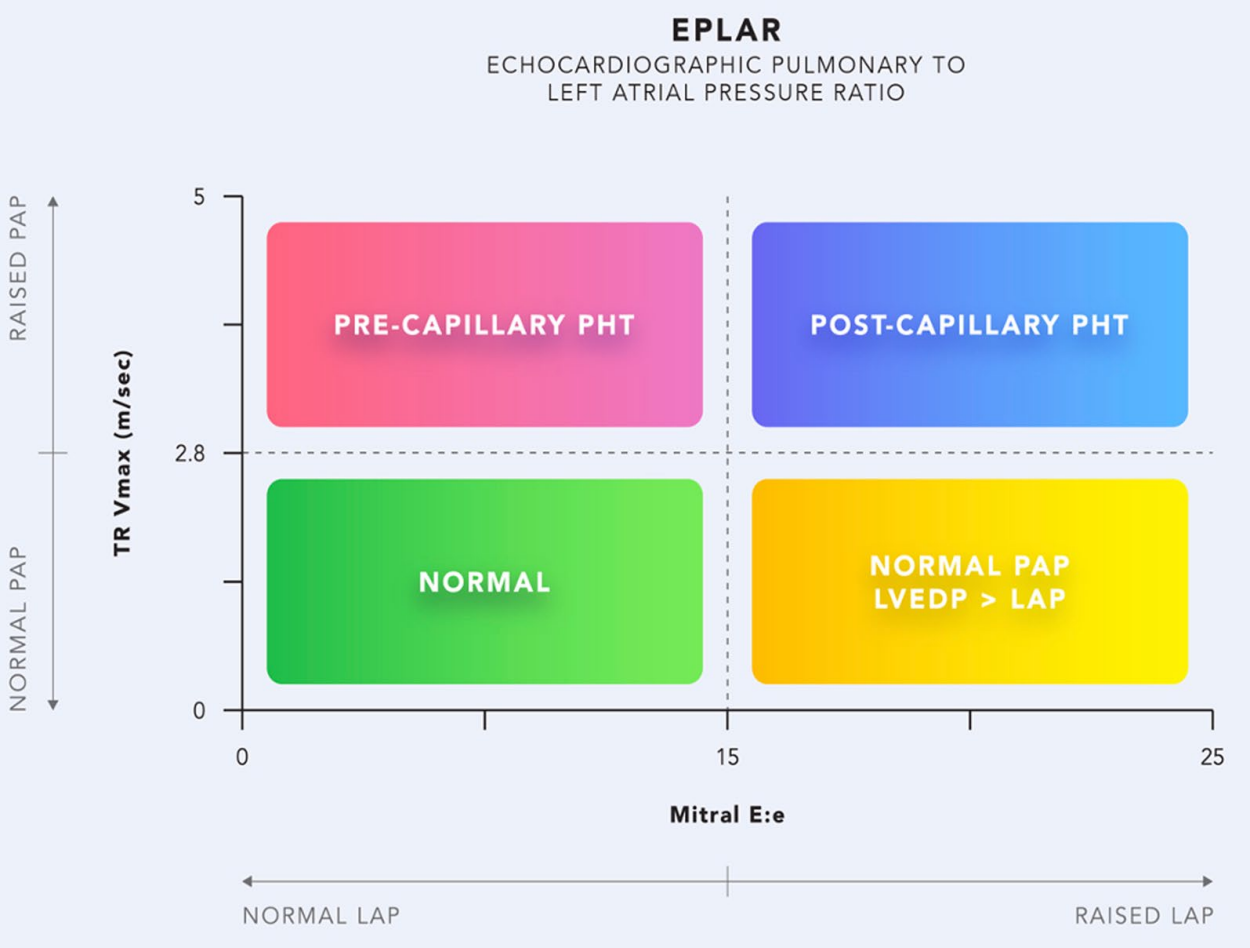
ePLAR = TRV/E/e′. Post-capillary pulmonary hypertension (PHT) is characterised by a lower ePLAR given E/e′ will be higher in these groups. Pre-capillary PHT with lower E/e′ has a higher ePLAR ratio. (A cut off value of < 0.28 m/s for post-capillary PH yielded 83% sensitivity and specificity, AUC 0.87) [12]. *TRVmax * tricuspid regurgitation maximum velocity, m/sec. *PAP* pulmonary artery pressure, mmHg

The impact of different PH haemodynamic subgroups on RV function is increasingly recognised [13]. A higher incidence of RV dysfunction and RV–pulmonary arterial uncoupling (measured by tricuspid annular planar systolic excursion (TAPSE)/systolic pulmonary artery systolic pressure (sPAP) ratio) was found in those with pre-capillary and combined pre- and post-capillary PH than in isolated post-capillary PH [14]. ePLAR (echocardiographic pulmonary-to-left atrial ratio using tricuspid regurgitant velocity and E/e′) appears to be a simple, non-invasive ratio in differentiating pre- and post-capillary PH with reasonable accuracy, albeit in non-critically ill cohorts [15] (Fig. 4). Patients with RV dysfunction coupled with a low/normal mean LAP and high pulmonary pressures may benefit from pulmonary vasodilators, e.g. nitric oxide. In contrast, those with a high mean LAP and isolated post-capillary PH may derive benefit from diuretics, and pulmonary vasodilators in this group may worsen pulmonary oedema [16]. These diverging treatment strategies emphasise the potential benefit of amalgamating LAP measurement into categorising RV–pulmonary circuit dysfunction. Further investigation of the feasibility and utility of ePLAR in critically ill patients with RV dysfunction would be of interest.

### Bedside methods for assessing LAP

#### Invasive: pulmonary artery occlusion pressure (PAOP)

The challenges in correlating PAOP, LAP and LVEDP when using a PA catheter have been subject to intense evaluation in previously published works [17, 18] and are summarised in Table 1. Table 2 summarises non-critical care studies investigating the correlation between the PAOP and LVEDP during left heart catheterisation (LHC) showing varying results [19–22]. Data comparing PAOP and LVEDP in critical care populations are scare and conflicting, and a tabulated summary is provided in Table 3 [23–25]. In 1974, Lozman et al. evaluated five ventilated post-operative cardiac surgical patients without ARDS and showed that the relationship between PAOP and directly measured LAP was lost at PEEP levels above 15 cm H20 [23]. Jardin et al. demonstrated that below a PEEP of 10cmH20, PAOP correlated with invasively measured LVEDP; however, this correlation was diminished at PEEP values > 10 [24]. Teboul et al. have shown that PAOP correlated strongly with invasively measured post-A wave LVEDP in patients with ARDS with PEEPs up to 20 cm H20. They suggested this observed correlation of PAOP and LVEDP is due to surrounding diseased lung preventing alveolar vessel compression [25].

**Table 1:** Caveats of invasive pulmonary artery catheter measurement of PAOP and correlation with LAP, LVEDP and LVEDV in critical illness

PAOP $\ne$ LAP $\ne$ LVEDP	LVEDP $\ne$ LVEDV
Technical, e.g. calibration, zeroing, damping, digital recording, respiratory variation	Altered LV chamber compliance, e.g. diastolic dysfunction, myocardial ischaemia, LV hypertrophy (chronic HTN, aortic stenosis, hypertrophic cardiomyopathy, cardiac amyloid)
Catheter tip position in non-west zone 3, ‘overwedging’	Increased pleural pressure (PEEP, mechanical ventilation)
Physiological non-west zone 3 (ARDS, hypovolaemia, low CO, high PEEP)	High juxtacardiac pressures (cardiac tamponade, constrictive pericarditis, PEEP)
Valvular disease (Mitral valve stenosis and regurgitation (meanLAP > LVEDP), Aortic regurgitation (meanLAP < LVEDP))	RV pressure/volume overload and leftward septal shift (PE, ARDS, RV infarction)
LA pathology (Atrial myxoma, reduced LA compliance (following ablation procedure, critical illness)	
Pulmonary venous obstruction (tumour, mediastinal fibrosis, extensive pulmonary venous thrombosis, pulmonary veno-occlusive disease)	

*PEEP* positive end-expiratory pressure; HTN-systemic hypertension; *LV* left ventricle; *RV* right ventricle; *PE* pulmonary embolism; *LA* left atrium; *ARDS* acute respiratory distress syndrome; *LVEDP* left ventricular end-diastolic pressure; *CO* cardiac output; *PAOP* pulmonary artery occlusion pressure; *LAP* left atrial pressure; *LVEDV* left ventricular end-diastolic volume

**Table 2:** Accuracy of LAP measured by non-invasive and invasive techniques in the non-critically ill

Studies	Population	Methods	Measurement	Main findings	Exclusion criteria
Non-critical care studies evaluating PAOP and invasive LVEDP					
Sato et al. [16]	Elective cardiac catheterisation	*N* = 79 Retrospective subgroup analysis of those undergoing simultaneous LHC and RHC	PAOP during RHC (method not specified) versus post-A wave LVEDP during LHC	Strong correlation, *r* = 0.82 *p* < 0.001	ACS, AF, mitral valve surgery, mitral valve disease (stenosis, severe regurgitation or severe MAC), severe AR, prior heart transplantation. Heart rate > 100, any change in diuretic, vasodilator or antihypertensive treatment between cardiac catheterization and echocardiography
Hemnes et al. [17]	PH	*N* = 2270 Retrospective, single-centre study over 16 yrs in patients referred for simultaneous RHC and LHC	Digitised mean PAOP during RHC and ‘manually measured’ LVDEP during LHC	Mean difference − 1.6 mmHg IQR − 15 to 12 mmHg Modest correlation by linear regression *r*^2^ = 0.36, *p* < 0.001 In those with PH (*n* = 1,331) mean difference 0.3 mmHg IQR − 14 to 14mmHG, less correlation *r*^2^ = 0.27, *p* < 0.001	Any patient deemed to have ‘extreme critical illness’. Acute decompensation, shock, vital signs suggesting imminent death) or cardiac-related critical illness (hypertensive crisis)
Halpern et al. [18]	PH	*N* = 11,523 Retrospective, single centre. Patients referred for simultaneous RHC and LHC data over a 10-year period	Mean LAP during RHC versus simultaneously measured LVEDP during LHC	Moderate discrimination between patients with high vs normal LVEDP AUROC = 0.84; 95% CI 0.81 to 0.86 PAOP poorly calibrated to LVEDP (Bland–Altman limits of agreement, − 15.2 to 9.5 mm Hg *N* = 3926 with mean PAP greater than 25 mm Hg. 14.8% with a PAOP < 15 mm Hg of which 310 (53.5%) were misclassified, having an invasive LVEDP > 15 mm Hg	Mitral stenosis or HR > 130 bpm
Mascherbauer et al. [19]	HFpEF	*N* = 152 Prospective simultaneous RHC and LHC	Digitised mean PAOP over 8 cardiac cycles during RHC LVEDP ‘manually measured’ during LHC	Modest pressure difference 2.0 ± 4.4 mmHg between PAOP and LVEDP	> Moderate valvular heart disease, congenital heart disease, significant coronary artery disease requiring PCI or CABG. Severe congenital abnormalities of the lungs, thorax, or diaphragm, COPD with a forced expiratory volume in 1 s (FEV1) < 50%
Non-critical care studies evaluating echo Doppler and invasive LVEDP or PAOP					
Lancelloti et al. [25]	Patients with and without heart failure (25% had an EF < 50%, 53% had coronary artery disease) clinically requiring coronary angiogram	*N* = 159 Prospective multicentre, 9 centres in Europe	Echo estimate of LVFP using 2016 recommendations (E/A, E/e′, left atrial volume index, tricuspid regurgitation jet velocity) within 30 min of LHC measured LVEDP (elevated defined as ≥ 15 mm Hg and measured as the mean LVEDP averaged over 3 consecutive cycles)	65% of patients with normal non-invasive estimate of LVFP had normal LVEDP. 79% of those with elevated non-invasive LVFP had elevated invasive LVEDP Sensitivity 75%, specificity 74%, PPV 39%, NPV 93%, AUC 0.78	ACS, > mild valvular heart disease, valvular prosthesis, MAC, previous MI involving basal septum and/or basal lateral wall, AF/severe arrhythmias precluding Doppler analysis, LBBB, PPM HCM, pericardial disease, inadequate echocardiographic imaging or any administration of diuretics or vasodilators within the day prior the hemodynamic evaluation
Balaney et al. [26]	‘Clinically indicated LHC’	*N* = 90. Prospective, single centre 9 patients ‘indeterminate’, total *n* = 81	Non-invasive estimate of LVFP using 2016 recommendations versus invasive LVEDP (pre-A pressure at end expiration with LHC)	Sensitivity (of the detection of elevated LVFP) 0.69, specificity 0.81, PPV 0.77, NPV 0.74, accuracy 0.75	Hemodynamically unstable, AF, > moderate mitral regurgitation, > moderate MAC, mitral stenosis, heart transplantation, sinus tachycardia, prosthetic valves
Nauta et al. [27]	HFpEF	Systematic review of 9 studies Comparison of E/e′ to invasively measured ‘LVFP’	Five studies used PAOP and four studies used LVEDP as invasive reference. Invasive measurements were simultaneous or directly after echo in seven out of nine studies	Meta-analysis using a random-effects model yielded a pooled *r* correlation coefficient of 0.56	101 full test articles assessed

*LHC* left heart catheterisation; *RHC* right heart catheterisation; *PPV* positive predictive value; *NPV* negative predictive value; *LVFP* left ventricular filling pressure; *LVEDP* left ventricular end diastolic pressure; *PAOP* pulmonary artery occlusion pressure; *HFpEF* heart failure with preserved ejection fraction; *ACS* acute coronary syndrome; *PH* pulmonary hypertension; *PAP* pulmonary artery pressure; *MAC* mitral annular calcification; *MI* myocardial infarction; *LBBB* left bundle branch block; *PPM* permanent pacemaker; *HCM* hypertrophic cardiomyopathy; *PCI* percutaneous coronary intervention; *CABG* coronary artery bypass grafting

**Table 3:** Studies evaluating non-invasive and invasive LAP assessment in critical care populations

Studies	Methods	Main findings	Exclusion
Invasive PAOP versus LVEDP studies			
Lozman et al. [22]	Single centre, *N* = 5. Invasively ventilated post-operative cardiac surgical patients without ARDS	The relationship between PAOP and directly measured LAP was lost at PEEP levels above 15 cm H20	Not specified
Jardin et al. [23]	Single centre, *N* = 10. Invasively ventilated patients with ARDS. PAOP was measured at end expiration. LVEDP was measured with an LV catheter, defined as the pre-ejection diastolic plateau or onset of the ECG q wave	Below PEEPs of 10cmH20, PAOP correlated with invasively measured LVEDP. Correlation was diminished at PEEP values > 10cmH20 with PAOP being higher than LVEDP. Correlation values not provided	Not specified
Teboul et al. [24]	Single centre, *N* = 12. Patients with ARDS. Simultaneous measurement of PAOP and LVEDP at PEEP levels up to 20cmH20. PAOP, measured as the mean value at end expiration and averaged over 5 or more cycles. LVEDP measured at the ‘z’ point (i.e. at the end of the ‘a’ wave)	PAOP usually agreed with invasively measured post-A wave LVEDP by 1–2 mmHg. ‘Close correlation’ was seen between PAOP and LVEDP at PEEP levels up to 20cmH20 Authors suggested this observed correlation of PAOP and LVEDP is due to surrounding diseased lung preventing alveolar vessel compression	Contraindication to left heart catheterisation (aorto-femoral atherosclerosis, aortic stenosis, thrombocytopenia or coagulopathy)
Non-invasive Echo Doppler LAP versus PAOP			
Brault et al. [29]	Prospective study across two ICUs. *N* = 98. All mechanically ventilated. Pooled analysis of 3 prospective cohorts with simultaneously assessed LAP by echo and PAOP by PA catheter measured at end expiration and averaged over 5 cardiac cycles	The sensitivity and specificity of ASE/EACVI guidelines for predicting elevated PAOP ≥ 18 mmHg were both 74%. Agreement between echocardiography measured raised LAP and elevated PAOP (> 18 mmHg) was moderate (Cohen’s Kappa, 0.48; 95% CI, 0.39–0.70) New simplified algorithm proposed: LVEF < 45% E/A cut off < 1.5 and LVEF > 45% lateral e’ cut off > 8 for predicting PAOP < 18 mmHg. Sensitivity and specificity of the proposed algorithm for predicting an elevated PAOP were 87% and 73%, respectively	Arrhythmia, severe mitral or aortic valvulopathy, merged Doppler mitral flow, or inadequate image quality for Doppler measurements
Vignon et al. [31]	Prospective, single-centre, two consecutive 3-year periods. *N* = 88 mechanically ventilated patients. Protocol A, *n* = 56 used to estimate Doppler parameters predicting PAOP ≤ 18 mmHg, Protocol B, *n* = 32, derived Doppler values from protocol A were tested prospectively	In protocol B, mitral E/A ≤ 1.4, pulmonary vein S/D > 0.65 and systolic fraction > 44% best predicted an invasive PAOP ≤ 18 mmHg. Correlations between Doppler and PAOP values were consistently closer in the subset of patients with depressed LV systolic function Lateral E/e′ ≤ 8.0 or E/Vp ≤ 1.7 predicted a PAOP ≤ 18 mmHg with a sensitivity of 83% and 80%, and a specificity of 88% and 100%, respectively. Areas under ROC curves of lateral E/e′ and E/Vp were similar (0.91 ± 0.07 vs 0.92 ± 0.07: *p* = 0.53)	Non-sinus rhythm, ‘relevant’ valvulopathy, AV conduction abnormality, TOE contraindication
Nagueh et al. [32]	Single-centre ICU. Complex design. *N* = 36 (20 mechanically ventilated) having adequate TTE Doppler tracings and PAC (initial study group). 32 patients were later enrolled (prospective study group, unspecified proportion mechanically ventilated)	Correlation of PAOP with E/A ratio (*r* = 0.75), IVRT (*r* = − 0.55), DT (*r* = − 0.5) and atrial filing fraction (*r* = − 0.65). PA occlusion pressure equation derived incorporating E/A and IVRT and correlation assessed with invasive PAOP: *r* = 0.79 and *r* = 0.88 in initial and prospective groups, respectively	AF, inadequate Doppler recording, fusion of E/A
Mousavi et al. [33]	Retrospective, single centre. *N* = 40 patients with septic shock. TTE Doppler and PAC PAOP within 4 h. Methods for PAOP measurement not specified	Correlation between average E/e′ and PAOP (*r* = 0 .84, *p* < 0.05)	Not fulfilling criteria for septic shock
Dokainish et al. [34]	Prospective, single-centre ICU. *N* = 50, patients who had existing PAC. 21 invasively ventilated. Simultaneous measurements of echo Doppler and PAOP and BNP	Correlation between E/e′ and PAOP: *r* = 0.69 (*p* < 0.001) E/e′ > 15 was the optimal cut off to predict PAOP > 15 mm Hg (sensitivity, 86%; specificity, 88%) E/e′ was more accurate in those with cardiac disease	AF, paced rhythm, severe MR, MS, mitral prosthesis, severe MAC, acute MI, unstable angina, and CABG within 72 h
Combes et al. [35]	Prospective, single-centre ICU. *N* = 23, consecutive mechanically ventilated patients. TOE or TTE Doppler versus PAOP by PA catheter	PAOP and the lateral E/e′ correlation (*r* = 0.84) and medial E/e′ correlation (*r* = 0.76). The sensitivities and specificities of estimating PAOP > 15 mmHg were, respectively, 86% and 81% for lateral E/e′ > 7.5 and 76% and 80% for medial E/e′ > 9	Age < 18 years, non-sinus rhythm, mitral insufficiency greater than grade 2 and mitral stenosis, prosthetic mitral valve, tachycardia that prevented a distinct separation between the E and A waves
Bouhemad et al. [36]	Prospective, single-centre ICU. *N* = 60, admitted with septic shock and acute lung injury Simultaneous comparison of echo Doppler with TOE and PAOP. All patients mechanically ventilated. PEEP was removed or reduced to 5cmH20 during study	Mean bias variation between invasive PAOP and PAOP measured with Doppler (using the equation 0.97x E/e′ + 4.34) was of 0.5 mmHg with a precision of 2.0 mmHg ROC curves demonstrated that an E/e′ > 6 was an accurate predictor of a PAOP of ≥ 13 mmHg (AUC 0.98) Changes in PAOP were significantly correlated to changes in E/e′ (Rho 0.84, *p* < 0.0001)	Unable to have TOE, lack of sinus rhythm, BBB, left ventricular systolic dysfunction, presence of a significant mitral pathology, CAD and segmental wall motion abnormality
Dabaghi et al. [37]	Prospective, single-centre ICU over 6-month period in consecutive patients requiring invasive haemodynamic monitoring and echocardiography. *N* = 49. PAOP performed at end expiration	Left ventricular filling pressure calculated non-invasively by: 46 − (0.22 − x IVRT) − (0.10 × AFF) − (0.03 × DT) − (2/[E/A]) + (0.05 × MAR) Mean values 21 ± 8 vs 20 ± 8 mm Hg, for non-invasive and invasive, respectively. Correlation *r* = 0.88	Not in sinus rhythm, MS or prosthetic mitral valve. PEEP was < 10cmH20 in all patients

*IVRT* isovolumic relaxation time; *AFF* atrial filling fraction; *DT* deceleration time; *MAR* time from the end of mitral flow to the R wave of the electrocardiogram; *Vp* flow propagation velocity by colour m mode Doppler; *BNP* brain natriuretic peptide; *MS* mitral stenosis; *MR* mitral regurgitation; *TOE* transoesophageal echo; *CAD* coronary artery disease; *ROC* receiver operating characteristic curves; *BBB* bundle branch block; *PEEP* positive end-expiratory pressure; *ARDS* acute respiratory distress syndrome; *ASE/EACVI* American Society of Echocardiography and the European Association of Cardiovascular Imaging. Other abbreviations as in Table 2

#### Non-invasive: echocardiography and Doppler techniques

**Fig. 5:**
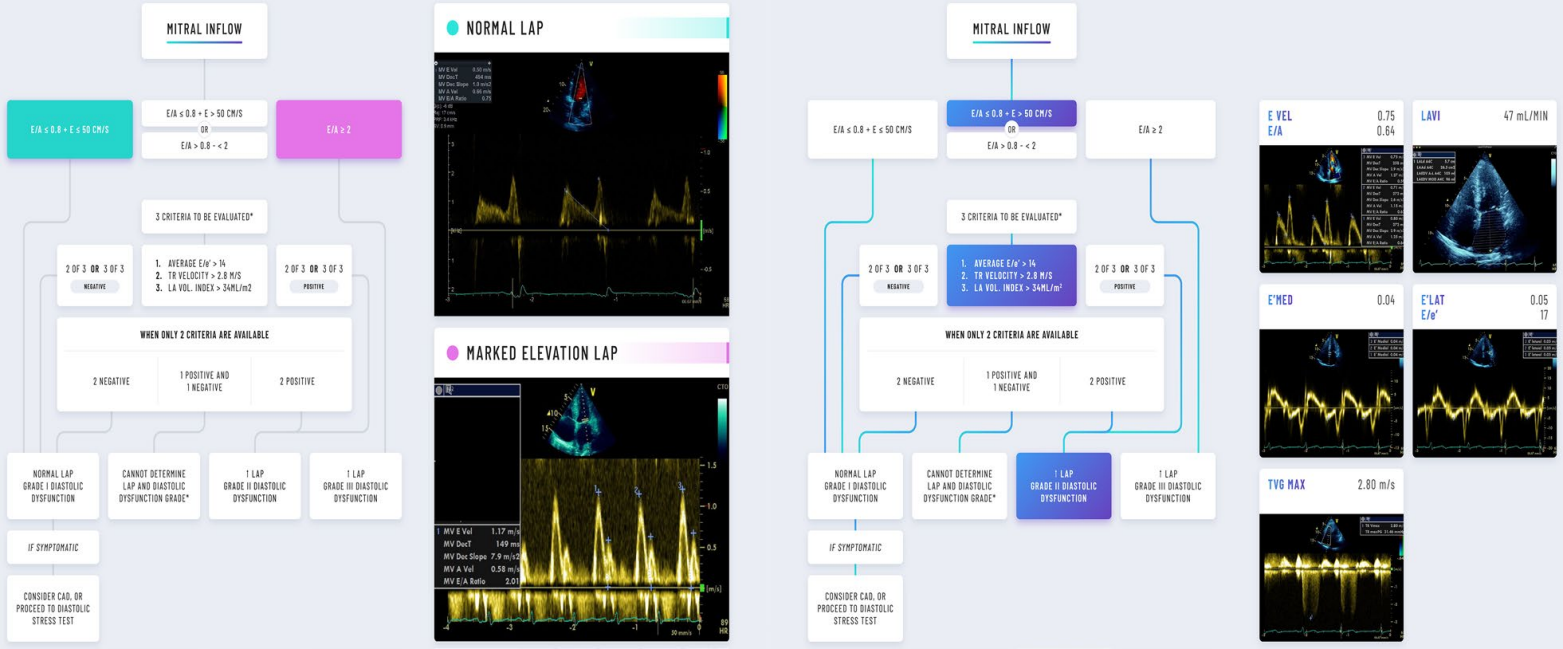
ASE/EACVI algorithms for estimating LAP in those with * reduced* left ventricular ejection fraction (EF) of < 50% (or normal EF with the presence of structural disease). Left panel, demonstrates where E/A ratio and E velocity, or E/A alone can differentiate normal versus elevated LAP in those with grade 1 and grade 3 diastolic dysfunction, respectively. Right panel, demonstrates a patient where 3 further criteria are required to decide if there is raised LAP: E/e′, TR Velocity and LA volume index (LAVI) showing a patient with grade 2 diastolic dysfunction and raised LAP

Investigation of LAP non-invasively using Doppler has been studied for over 30 years [26]. The most recent 2016 American Society of Echocardiography and the European Association of Cardiovascular Imaging (ASE/EACVI) guidelines estimate mean LAP through Doppler assessment of diastolic blood flow between the left atrium and left ventricle (mitral E to A wave ratio), tissue Doppler imaging (TDI) of the mitral annulus, the tricuspid regurgitant flow velocity and LA volumes as shown in Fig. 5 [27]. Importantly for the critical care physician who is interested in presence of raised LAP for treatment decisions, these guidelines began to differentiate between the two major objectives—LV diastolic dysfunction and LAP (Fig. 5).

The Euro-Filling study enrolled 159 patients in 9 centres, comparing non-invasive LAP measurements using ASE/ESCVI guidelines with invasive measurements of LVEDP. Of those with a normal non-invasive LAP, only 65% had a normal invasive LVEDP. Of those with an elevated non-invasive LAP, 79% had elevated invasive LVEDP. Overall, the sensitivity was 75% and specificity 74% giving a PPV of 39% and NPV 93% with an AUC of 0.78 [28]. In a similar study, 90 patients undergoing invasive cardiac catheterisation underwent TTE immediately prior to the procedure. The non-invasive LAP was accurate, when compared to the pre-A invasive measurement in 75% and inaccurate in 25%. In the latter group, the non-invasive LAP was overestimated in 8/20 and underestimated in 12/20 [29]. In these and other studies, the use of a single parameter as opposed to the guidelines use of 4 parameters was found to be highly inaccurate. Even the favoured one of E/e′ delivered only a moderate correlation to invasive measurements. Once again, these studies do not include the type of patients commonly found in the critical care setting who often have cardiac pathologies other than coronary artery disease. A tabulated summary is provided in Table 2 [28–30].

It is of little surprise that pathophysiology unique to critical illness (mechanical ventilation, vasoactive agents, fluid shifts) can make application of these algorithms more challenging [31]. Brault et al. [32] who compared ASE/EACVI echo Doppler LAP algorithms to PAOP (measured at end expiration and averaged over five non-consecutive cardiac cycles) in 98 mechanically ventilated patients found a sensitivity and specificity of 74% for ASE/EACVI algorithms to predict elevated PAOP ≥ 18 mmHg. Agreement between echocardiography and PAOP was moderate (Cohen’s Kappa, 0.48; 95% CI, 0.39–0.70). Overall, the guidelines show better discriminatory performance in the critically ill than previous iterations as shown by Clancy et al. [33] and offer an unrivalled framework in our patient group. Table 3 provides a tabulated summary of critical care studies that have compared echo Doppler LAP to PAOP values in critically ill patients [32, 34–40].

The ratio of early diastolic mitral inflow to average mitral annular tissue velocity (E/e′) has been most extensively studied in the cardiology population [28, 30] and has gained some interest in the critical care literature [32, 34, 36–39]. E/e′ is less load dependent and can be used to assess for raised LAP in those with atrial fibrillation (AF), making it a favoured choice in critical care. A septal E/e′ of > 11, as well as lack of mitral E velocity beat to beat variation, are suggestive of raised LAP in AF [27].

As with any haemodynamic measurement, the use of a single parameter to evaluate LAP should be avoided, and E/e′ is no exception [19]. Although a normal E/e′ does not rule out high LAP, an E/e′ > 15 does have a high specificity in identifying a high LAP [41]. This is perhaps of greatest pragmatic benefit when decisions on further fluid resuscitation are needed at the bedside: an E/e′ > 15 in this scenario would strongly favour a patient with ‘fluid intolerance’. At the other extreme, a low lateral E/e′ of < 8 has shown good diagnostic accuracy to predict PAOP < 18 mmHg [34].

A further challenge of the algorithm to identify patients with high LAP in critical care is the inability of the LA to dilate acutely (in comparison to the right atrium) [42]. Critically ill patients can have acutely high LAP despite a normal LA size, for example, those with volume overload or sepsis and acute diastolic dysfunction [3]. In summary, a dilated LA (LA volume index (LAVI) ≥ 34mls/m^2^) should raise suspicion for raised LAP, but a normal LA size should not exclude raised LAP. Echocardiographic evaluation of the interatrial septal (IAS) kinetics throughout the respiratory cycle may add pertinent information. Patients with fixed bowing of the IAS to the right are more likely to have raised LAP [43]. Considering right atrial pressure is important however given it is the relative pressure difference between the atria that determines position of the interatrial septum. Additional parameters, including a reduced E wave deceleration time (< 160 ms), alterations in the pulmonary venous Doppler waveform such as a reduced contribution to left atrial filling during systole (S/D ratio < 1), reduced isovolumetric relaxation time (IVRT) of < 60 ms and a mitral ‘L’ wave of > 20 cm/sec, may be additive in identifying raised LAP. An appraisal of their merits and disadvantages is discussed by Nagueh et al. [27].

**Fig. 6:**
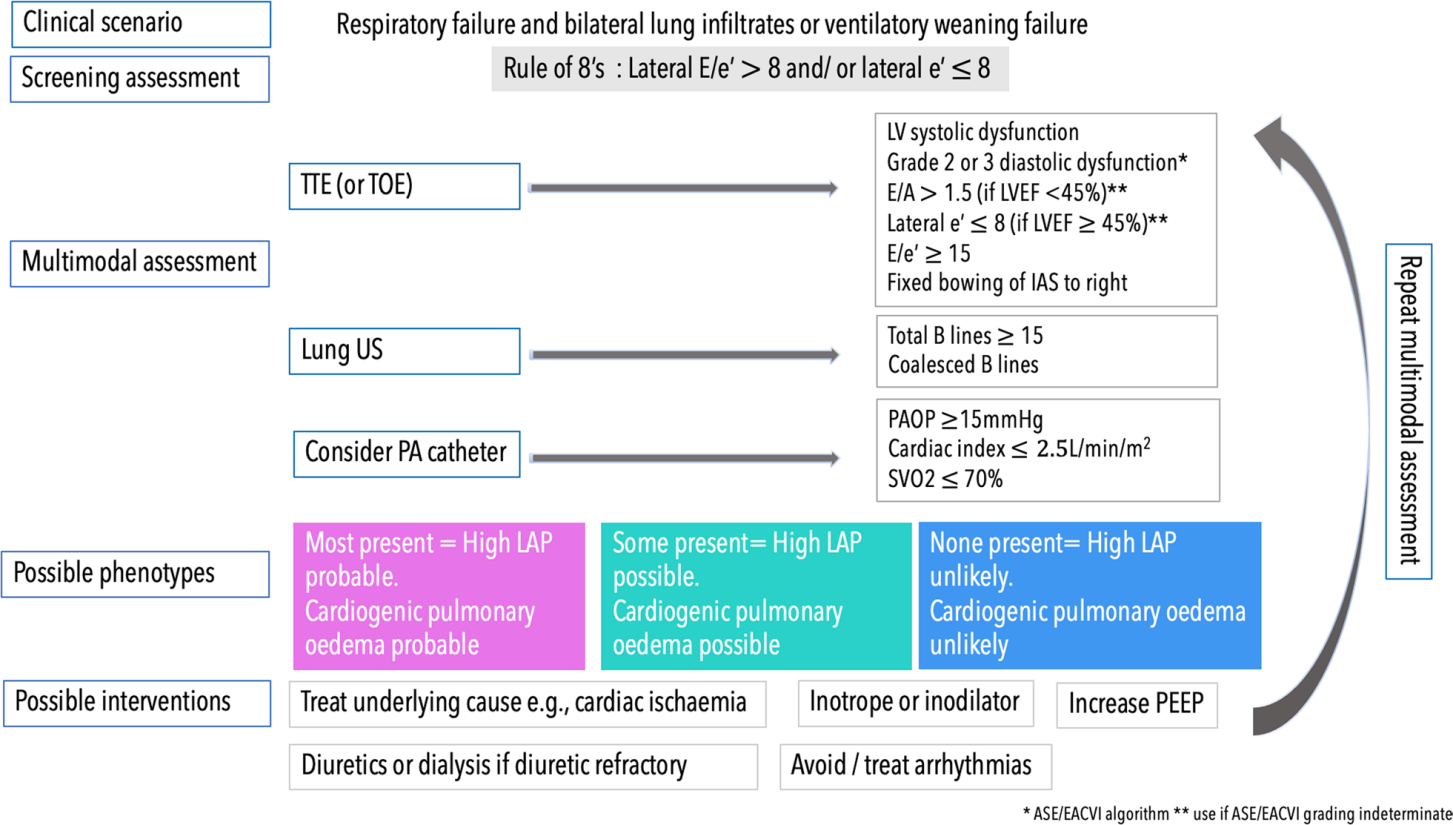
Proposed multimodal algorithm for a patient presenting with acute hypoxic respiratory failure or failing to wean from mechanical ventilation. Methods for assessment of LAP and its upstream consequence of cardiogenic pulmonary oedema as well as targeted treatment options suggested. * [24], **[29]

Overall, when it comes to LAP measurement there exists a lack of uniformity in methods and ‘what’ is being measured. These issues are further compounded by the heterogeneity of the populations included. We shouldn’t be too hasty however in abandoning LAP measurement at the bedside altogether. A non-invasive, rapid beside screening tool to identify patients with possible raised LAP could be ‘the rule of 8’s’: lateral E/e′ > 8 [34] and a lateral e’ ≤ 8 cm/s [32]. This tool could serve as a trigger to temporarily halt further fluid resuscitation and instigate multimodal assessment of cardiopulmonary performance as proposed in Fig. 6.

#### Newer non-invasive measurements of LAP: LA strain and left atrial expansion index

**Fig. 7:**
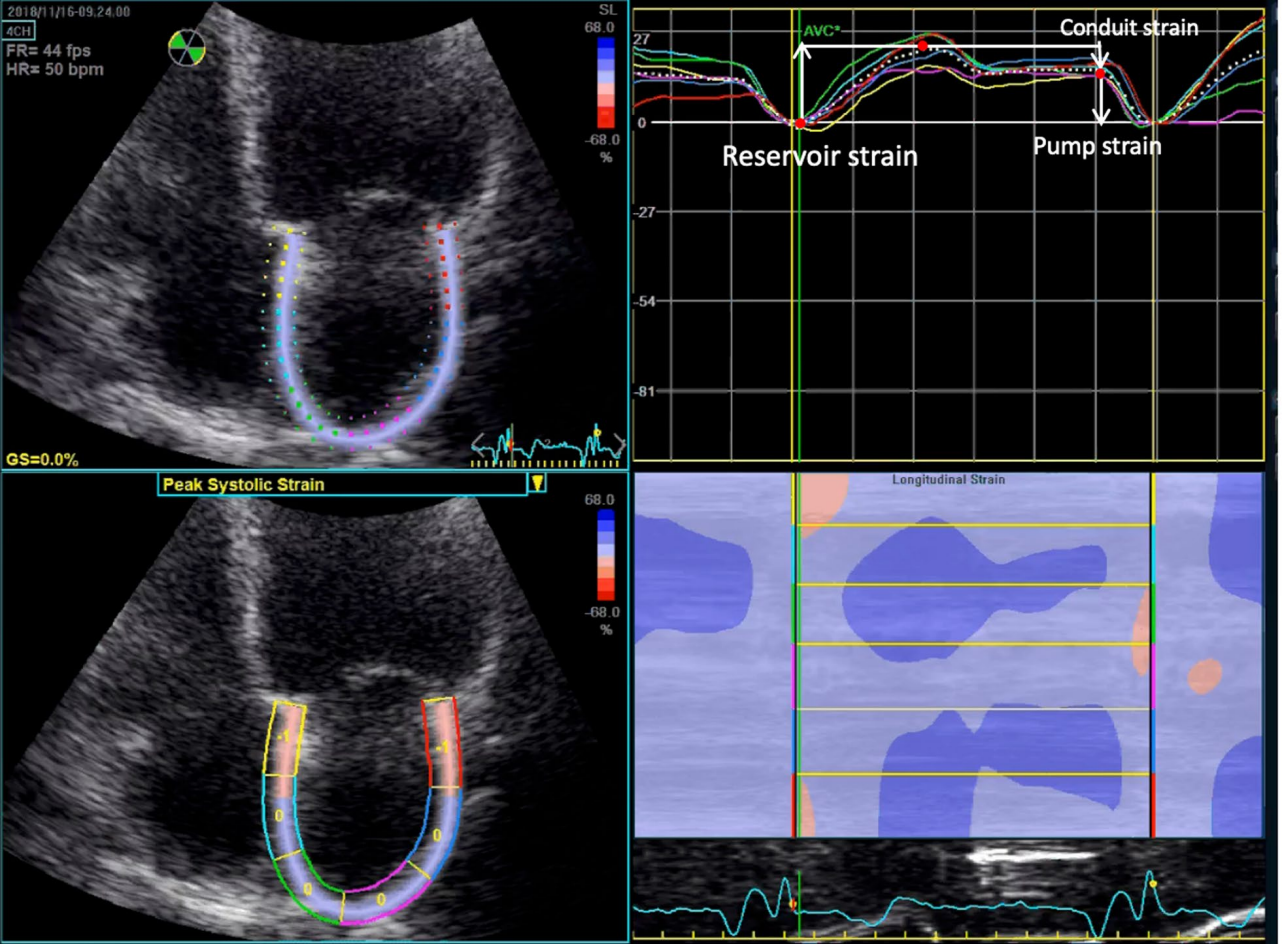
LA strain using non-foreshortened A4C LA views. White dashed strain curve showing average values of 6 segments. Ventricular end-diastole is recommended as the time reference to define the zero-baseline for strain curves. As depicted by the white arrows: LA reservoir strain = difference of the strain value at mitral valve opening minus ventricular end-diastole. LA conduit strain = difference of the strain value at the onset of atrial contraction minus mitral valve opening. LA pump strain = difference of the strain value at ventricular end-diastole minus onset of atrial contraction [44]

LA strain uses angle independent speckle tracking imaging to assess LA function and stiffness [44]. The increased participation of LA contraction to end-diastolic LV filling is increased in the presence of LV diastolic dysfunction, up to the point when LA is failing because of excessive LVEDP. Studies have demonstrated an inverse relationship between LA global strain and LV end-diastolic pressures [45]. LA strain should be measured using a non-foreshortened apical-4-chamber (A4C) view of the LA where values of LA strain for reservoir, conduit and pump functions are measured [46] (Fig. 7). Inoue et al. evaluated 322 patients referred for left or right heart catheterisation in a multicentre study [47]. Cut off values for LA reservoir strain of < 18% and LA pump strain of < 8% had an AUC of 0.76 and AUC 0.77, respectively, for detecting increased LVFP (defined as PAOP > 12 mmHg or LVEDP > 16 mmHg). LA strain didn’t perform well in predicting LVFP in those with AF. These values have been proposed to serve as substitute parameters for those with missing criteria in ASE/EACVI algorithms that would otherwise be classified as ‘indeterminate’ (Fig. 5), providing there are no exclusion criteria (AF, mitral valve disease, and left bundle branch block amongst others) [48].

There has been increasing interest in the utility of the relative left atrial volume change over the cardiac cycle to predict filling pressure. The hypothesis being that a smaller volume expansion of the LA between systole and diastole predicts higher LAP. The value, expressed as a percentage, is known as the left atrial expansion index (LAEI) and is calculated by the formula: (Volmax − Volmin) × 100%/Volmin, where Volmax = maximal LA volume and Volmin = minimal LA volume. Genovese et al. investigated its use in over six hundred patients with chronic cardiac disease [49]. A reasonable linear correlation was found between logarithmically transformed LAEI and PAOP (*r* = 0.73, *p* < 0.001). Whilst LA strain and LAEI have shown promise in the cardiology setting, prospective data are needed to assess their role in the critical care arena.

#### LAP and the ‘diastolic stress test’ of critical care

Patients may have pre-existing diastolic dysfunction or develop de novo diastolic dysfunction because of critical illness such as sepsis [4]. An important concept to appreciate is that the ‘diastolic stress test’ of critical illness can shift patients from a normal ‘resting’ LAP to a high LAP state, with corresponding increases in E/e′ ratio. This is because the mitral annular velocity (e’) of the stiff left ventricle cannot increase to match the increased mitral E velocity as occurs with increased cardiac output demand [27]. This can be particularly problematic during ventilatory weaning leading to Weaning-induced Pulmonary Oedema (WiPO). There is no validated cut off E/e′ value to predict WiPO, though higher values are associated with increased risk of weaning failure. The reader is directed to detailed review of this topic elsewhere [50, 51]. Repeated echocardiographic assessment with an approach as outlined in Fig. 6 could help identify those at risk of WiPO and help guide treatment strategies. For example, patients with elevated LAP may benefit from more aggressive diuresis, higher levels of PEEP and a planned extubation to non-invasive ventilation.

#### LAP and acute respiratory distress syndrome (ARDS): the ‘grey zone’ patient

A PAOP ≥ 18 mmHg was a commonly accepted criterion to define cardiogenic oedema in ARDS [52]. However, it was not a ‘hard’ value, was seldom measured and it was increasingly appreciated that raised LVFP could coexist with ARDS, hence it was removed from revised diagnostic criteria [53]. Authors of the guidelines highlight the ongoing complexities in differentiating cardiogenic from non-cardiogenic pulmonary oedema and describe scenarios [53].

An elderly patient with chronic obstructive lung disease and congestive cardiac failure, with a central venous pressure of 15 mmHg and fulfilling ARDS criteria, is described as *probably* having an overlap of cardiogenic and non-cardiogenic pulmonary oedema [53]. In contrast, a multi-trauma patient fulfilling ARDS criteria with a small, hyperdynamic LV without pericardial effusion is likely to have non-cardiogenic pulmonary oedema ARDS. The latter scenario highlights the benefit of incorporating echocardiography, however, it describes the extreme ends of both echocardiographic and clinical spectrums where treatment decisions are often easier.

Unfortunately, many of our patients, like the elderly patient described with pre-existing cardiorespiratory comorbidity, fall into a ‘grey zone’ and there is frequent overlap of cardiogenic and non-cardiogenic aetiologies [54]. Ray et al. studied over 500 elderly patients presenting to the emergency department with acute respiratory failure and showed that those with cardiogenic pulmonary oedema had the highest mortality at 21%. Importantly, around one third of the total cohort was deemed to have inappropriate early treatment and this was associated with a doubling of in hospital mortality [54]. Early detection of raised LAP and lung oedema to prevent inappropriate therapy therefore is a key goal.

#### LAP and multimodal assessment: combining lung ultrasound

Lung ultrasound (US) assessment of B lines is relatively quick and can be used to identify pulmonary oedema [55, 56]. B lines can be seen in other non-cardiogenic lung oedema states particularly relevant to our population (interstitial syndrome of ARDS, pulmonary fibrosis) [56], hence the need to contextualise echocardiographic and clinical findings [57]. B line quantification methods have shown good diagnostic accuracy against extra vascular lung water impedance techniques in critically ill patients [58, 59]. A simplified 4-sector method described by Mayr et al. only just underperformed against the more laborious 28-sector method, and a cut off value of ≥ 15 B lines resulted in a sensitivity of 91.7% and specificity of 92.1% to identify patients with increased extravascular lung water (AUC 0.978) [59]. Counting the number of B lines can be difficult in those with coalesced lines and lung US scores evaluating the percentage of B lines occupying the pleural line may be better, however, the time required for post processing and offline analysis limits its application at the bedside for most users at this time [58]. Furthermore, in the shocked patient with echocardiographic LAP parameters falling into the ‘grey zone’, the finding of a predominant A line pattern, that is highly specific for a low/normal PAOP, can increase confidence that repeating a fluid challenge is unlikely to result in pulmonary oedema [60].

Time critical decisions on haemodynamic resuscitation often centre on ‘fluid tolerance versus intolerance’, as opposed to definitive diagnosis, and we often need rapid, yet rich haemodynamic information. Tempered with an awareness of the caveats, trends in LAP coupled to the upstream hydrostatic consequence using lung US could provide this information (proposed in Fig. 6).

## Conclusion

Located in a pivotal position in the journey of blood flowing from the right heart to the left ventricle, the contribution of left atrium to the circulation needs to be considered from a variety of perspectives. Seen as either a downstream station for the pulmonary blood flow, or an upstream one for filling of the left ventricle, the value of LAP to our haemodynamic armamentarium should not be underestimated. Currently utilised tools in evaluating LAP at the bedside, namely the PA catheter and Echo Doppler and 2D techniques, although having recognised technical drawbacks can be of benefit clinically if utilised correctly.

The combined strength of invasive and advanced echo techniques offers a pathway to evaluate both ends of the circuit, from LA function and pressure to pulmonary haemodynamics and RV function. Perhaps, this amalgamation can enable a more comprehensive understanding of ‘transpulmonary circuit dysfunction’ and its consequence to cardiac performance at the bedside, where it really matters.

## Data Availability

The data and material used in this article belong to the corresponding author and can be accessed with permission.

## Abbreviations

LAP: Left atrial pressure
LVEDP: Left ventricular end-diastolic pressure
PAOP: Pulmonary artery occlusion pressure
PVR: Pulmonary vascular resistance
RVD: Right ventricular dysfunction
RV-PA: Right ventricular–pulmonary artery
ePLAR: Echocardiographic pulmonary-to-left atrial ratio
PASP: Pulmonary artery systolic pressure
TAPSE: Tricuspid annular planar systolic excursion
